# Transient Lesion in the Splenium of the Corpus Callosum in Acute Uncomplicated Falciparum Malaria

**DOI:** 10.4269/ajtmh.13-0665

**Published:** 2014-06-04

**Authors:** Jiraporn Laothamatas, Christina L. Sammet, Xavier Golay, Marc Van Cauteren, Varinee Lekprasert, Noppadon Tangpukdee, Srivicha Krudsood, Wattana Leowattana, Polrat Wilairatana, Srirama V. Swaminathan, Robert L. DeLaPaz, Truman R. Brown, Sornchai Looareesuwan, Gary M. Brittenham

**Affiliations:** Department of Radiology, Faculty of Medicine Ramathibodi Hospital, Bangkok, Thailand; Ann and Robert H. Lurie Children's Hospital of Chicago, Chicago, Illinois; Institute of Neurology, University College London, London, United Kingdom; Philips Healthcare, Best, The Netherlands; The Hospital for Tropical Diseases, Faculty of Tropical Medicine, Mahidol University, Bangkok, Thailand; Departments of Radiology and Pediatrics and Medicine, Columbia University College of Physicians and Surgeons, New York, New York; Department of Radiology and Radiological Science, Medical University of South Carolina, Charleston, South Carolina

## Abstract

Patients with acute uncomplicated *Plasmodium falciparum* malaria have no evident neurologic disorder, vital organ dysfunction, or other severe manifestations of infection. Nonetheless, parasitized erythrocytes cytoadhere to the endothelium throughout their microvasculature, especially within the brain. We aimed to determine if 3 Tesla magnetic resonance imaging studies could detect evidence of cerebral abnormalities in these patients. Within 24 hours of admission, initial magnetic resonance imaging examinations found a lesion with restricted water diffusion in the mid-portion of the splenium of the corpus callosum of 4 (40%) of 10 male patients. The four patients who had a splenial lesion initially had evidence of more severe hemolysis and thrombocytopenia than the six patients who had no apparent abnormality. Repeat studies four weeks later found no residua of the lesions and resolution of the hematologic differences. These observations provide evidence for acute cerebral injury in the absence of severe or cerebral malaria.

## Introduction

Worldwide, the most important parasitic disease infecting the central nervous system is *Plasmodium falciparum* malaria.^1^ Of the five protozoan species of the genus *Plasmodium* that infect humans, *P. falciparum* causes almost all severe disease and death. *P. falciparum* is also the only species that modifies the erythrocyte surface so that infected erythrocytes are sequestered within the microvasculature by binding to the vascular endothelium.^2^ Cytoadherance of erythrocytes in vital organs throughout the body, especially in the brain, is believed to occur in all cases of falciparum malaria.^3^–^5^ Cerebral malaria is the most lethal complication of falciparum infection, presenting as a diffuse symmetric encephalopathy with alterations in the level of consciousness, ranging from drowsiness to deep coma, at times precipitated by seizures.^6^ In cerebral malaria, microvascular obstruction by sequestered parasitized erythrocytes leading to axonal damage has been proposed as a principal pathway responsible for coma and neurologic dysfunction, possibly in concert with a variety of immunopathologic mechanisms.^1^,^7^–^10^ Sequestration and cerebral damage have been documented at autopsy in patients who died of cerebral or other forms of severe malaria.^3^,^11^–^13^

In children in Malawi with cerebral malaria, magnetic resonance imaging (MRI) studies at 0.35 Tesla have identified distinctive findings in cortical, deep gray, and white matter structures.^14^,^15^ Acute uncomplicated falciparum malaria is an illness with *P. falciparum* asexual parasitemia and symptoms similar to those of a minor systemic viral infection, including headache, fever, chills, malaise, abdominal discomfort, and muscle and joint aches, but with no apparent neurologic disorder, vital organ dysfunction, or other severe clinical or laboratory manifestations of infection.^16^ Sequestration of parasitized erythrocytes within the microcirculation of the brain potentially develops in acute uncomplicated falciparum malaria^10^ but neuroimaging observations have been lacking.^17^ We aimed to determine if high-field (3.0 Tesla) MRI studies could detect evidence of cerebral abnormalities in adult patients with acute uncomplicated falciparum malaria in Thailand.

## Materials and Methods

### Study participants.

This study was a single-site prospective examination of adult patients with acute uncomplicated falciparum malaria admitted to a hospital in Thailand specializing in the care of patients with malaria. Acute uncomplicated falciparum malaria was defined as a febrile symptomatic illness with asexual *P. falciparum* parasitemia in the absence of any of the clinical features or laboratory findings meeting the World Health Organization criteria for severe malaria.^18^,^19^ This study was approved by the Institutional Review Boards of the institutions involved. A detailed verbal and written explanation of the research project was provided and each participant gave fully informed, signed consent to participate in the study. Patients were excluded from the study if there was a history of previous malarial infection, underlying disorders, seizures, splenectomy, drug or alcohol abuse, an age < 18 years or > 65 years, or if they were women who were or could be pregnant. To protect vulnerable populations, patients with a history of treatment for mental illness, imprisonment, or institutionalization were also excluded.

### Study procedures.

At admission, a history was obtained and a physical examination, including a standard neurologic evaluation, was performed, and the level of consciousness of each patient was assessed by using the Glasgow coma scale.^18^ After clinical evaluation and examination of thick and thin blood smears to establish the diagnosis, blood samples were obtained for hematologic and biochemical studies at baseline and periodically thereafter during the four-week hospitalization period. Hematologic studies were performed by using an Advia 120 Hematology Analyzer (Bayer HealthCare, Diagnostics Division, Tarrytown, NY).

All patients received antimalarial treatment with artemesinin combination therapy as part of clinical studies examining regimens for treatment of falciparum malaria and remained in the hospital for four weeks to assess clinical outcome, safety, and tolerance and to evaluate the cure rate at 28-day follow-up. Patients found to be co-infected by asexual forms of *P. vivax* were treated with the hospital's standard regimen for vivax malaria: chloroquine (30 mg base/kg given over 3 days) and primaquine (15 mg once a day for 14 days).

Parasite counts were determined by microscopy and counting infected erythrocytes per 1,000 erythrocytes in thin blood films, or calculated from the parasite count per 200 leukocytes in thick blood films. Parasite clearance time was defined as the time from the start of treatment until the patient's first negative blood film, with the blood film then remaining negative for 24 hours. Fever clearance time was defined as the time from the start of treatment until the oral temperature decreased below 37.5°C and then remained at or below this level for 48 hours.

### MRI techniques and analysis.

Within 24 hours of admission, MRI studies were performed by using a 3.0-Tesla scanner (Philips Achieva; Philips Healthcare Best, The Netherlands) with a phased-array multi-channel head coil and standard MRI pulse sequences to provide anatomic, clinical, and metabolic data. In addition to a standard neuroradiologic protocol, the studies included a T2-weighted fluid-attenuated inversion recovery (T2FLAIR) sequence (TR = 11000 ms, TE = 100 ms, TI = 2800 ms, FOV = 240 × 192 mm, acquisition matrix = 256 × 164, and recon matrix = 512 × 512); and a diffusion-weighted imaging echo-planar imaging (DWI-EPI) sequence (TR = 3000 ms, TE = 88 ms, b = [0,1000] s/mm^2^, FOV = 240 × 240 mm, acquisition matrix = 112 × 90, and recon matrix = 256 × 256). Using the diffusion-weighted images, maps of the apparent diffusion coefficient (ADC) were generated. The MRI studies were repeated near the end of the four-week hospitalization. All MRI studies were independently reviewed by two clinical radiologists for structural lesions.

### Statistical analysis.

Groups of patients were compared by using the unpaired Student's *t* tests for continuous variables with a Gaussian distribution, the Mann-Whitney test for nonparametric tests of continuous variables without a Gaussian distribution, and Fisher's exact test for proportions. Because the distributions of initial parasite counts were skewed, parasite counts were log-transformed before comparing the means with Student's *t* test. The results were then retransformed into antilogarithms to recover the original units and were expressed as geometric means with the upper and lower 95% confidence intervals.

## Results

**Figure 1.**
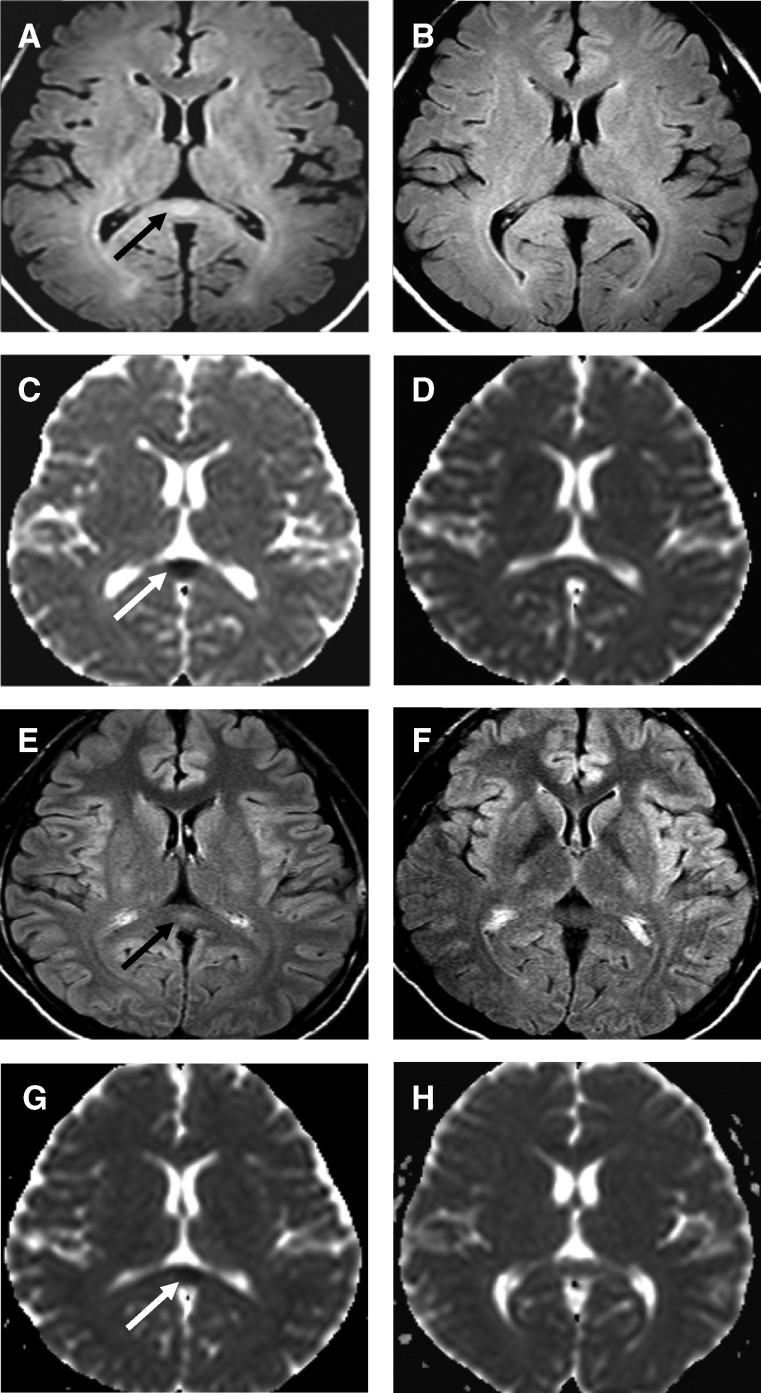
Magnetic resonance imaging studies of two patients with acute malaria who had no neurologic symptoms or signs. **A**–**D**, A 30 year-old man with uncomplicated malaria and a mixed infection (*Plasmodium falciparum*: 9,200 parasites/μL; *P. vivax*: 128 parasites/μL). **E**–**H**, A 22 year-old man with hyperparasitemia (*P. falciparum*: 588,000 parasites/μL). T2-weighted fluid-attenuated inversion recovery sequences (**A** and **E**) obtained shortly after admission show hyperintense, symmetrical oval lesions in the midline of the splenium of the corpus callosum (**black arrows**). Repeat examinations four weeks later (**B** and **F**), show resolution of the lesions. Diffusion-weighted imaging echo-planar imaging studies (**C** and **G**) shortly after admission showed relative decreases in the apparent diffusion coefficient within the lesions (**white arrows**) that had also resolved on the repeat studies (**D** and **H**) four weeks later.

**Figure 2.**
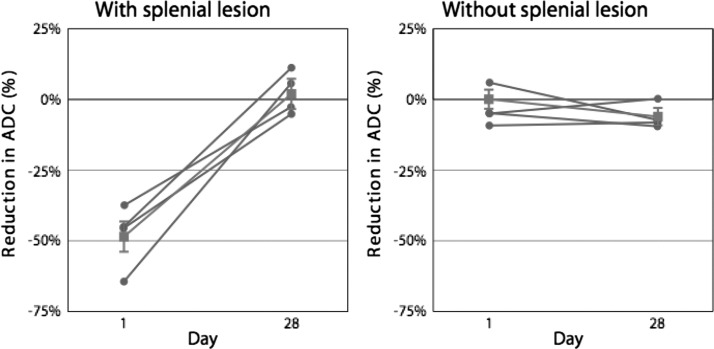
Change in apparent diffusion coefficient (ADC) (%) from day 1 (the first day after admission) to day 28 for four patients with a splenial lesion (**left panel**) and six patients without a splenial lesion (**right panel**). Gray circles and lines show the individual values; gray squares and lines show medians for each group of patients. Upper and lower 95% confidence intervals for the median are shown by the vertical gray lines.

We describe the first 10 consecutive patients with acute uncomplicated malaria who were examined with a 3.0-Tesla MR scanner. Their clinical characteristics are summarized in Table 1 . All patients were men 18–45 years of age. At admission, nine patients had only *P. falciparum* parasitemia, and one patient had a mixed infection with *P. vivax*. All patients were conscious and had a Glasgow coma score of 14–15. No patient had a history of coma or brain injury. No neurologic abnormalities were identified by standard examination. In 4 of the 10 patients (40%), the initial magnetic resonance study showed a hyperintense, symmetrical oval lesion in the midline of the splenium of the corpus callosum on images derived from T2FLAIR sequences (Figure 1

). Using maps of the ADC of water derived from the diffusion-weighted echo-planar imaging studies, we found that measurements of the corresponding regions of interest within the mid portion of the splenium of the corpus callosum showed a decrease in the ADC for each patient in whom a lesion was seen on the T2FLAIR examination. In this group of four patients, comparisons of the ADC estimates with those derived from comparable areas in the remaining six patients indicated a significant reduction in diffusion (Figure 2

) (*P* < 0.0001). Visual inspection of the ADC maps found no other focal abnormalities. Follow-up examinations at the end of the 28-day hospitalization found resolution of the splenial lesions on T2FLAIR images in the four patients initially affected. In addition, measurements of corresponding areas of the ADC maps now found no significant difference between the four patients initially affected and the remaining patients (Figure 2) (*P* > 0.11).

Quantitative assessment of the apparent diffusion coefficients (ADC) found an approximately 50% overall reduction in tissue water diffusion rates in the lesion on the initial MR study with recovery to normal four weeks later (Figure 2A). Further analysis of the ADC data showed that diffusion anisotropy was preserved in the lesion. The diffusion rate was approximately four times greater in the transverse direction (left–right, X), parallel to the crossing axons, than in the anterior-posterior direction (front–back, Y) or in the superior-inferior direction (head–toe, Z) (Figure 2B). In the acute phase MRI, diffusion appears to be equally proportionally restricted by approximately 50% in the X, Y, and Z axes but the magnitude of restriction is greatest by approximately four times in the X direction, parallel to the long axis of the crossing axons.

**Figure 3.**
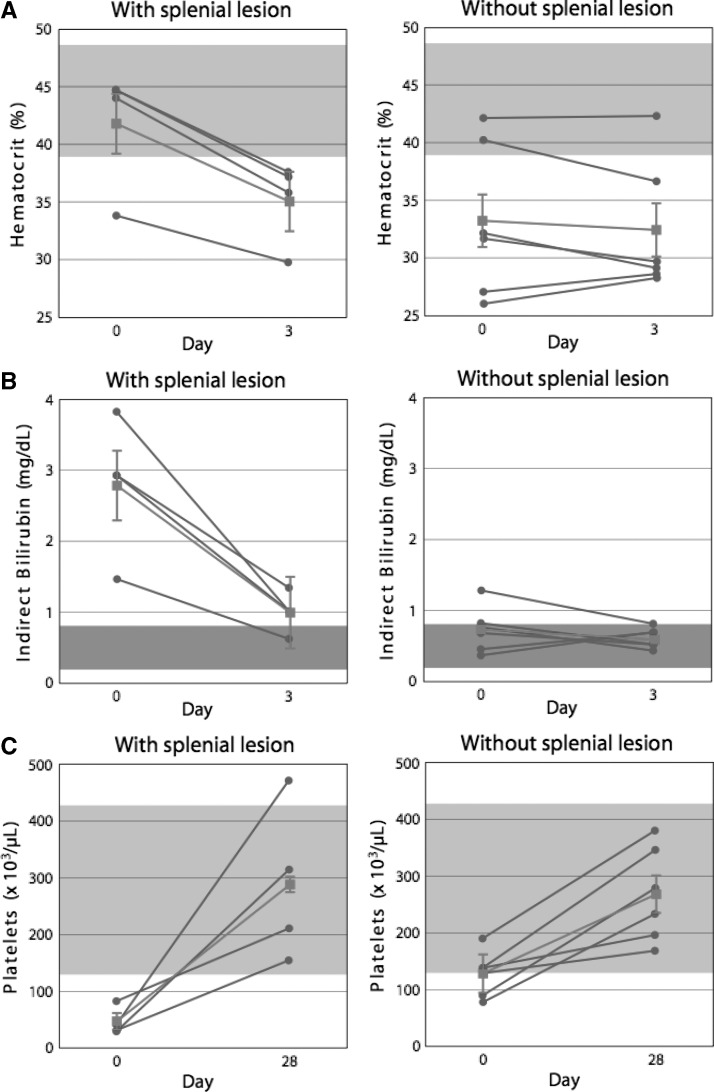
**A**, Change in hematocrit (%) from day 0 (admission) to day 3 for 4 patients with a splenial lesion (**left panel**) and six patients without a splenial lesion (**right panel**). Gray circles and lines show individual values; gray squares and lines show medians for each group of patients. Upper and lower 95% confidence intervals for the median are shown by the vertical gray lines. **B**, Change in indirect bilirubin concentration (mg/dL) from day 0 (admission) to day 3 for 4 patients with a splenial lesion (**left panel**) and 6 patients without a splenial lesion (**right panel**). Gray circles and lines show individual values; gray squares and lines shows medians for each group of patients. Upper and lower 95% confidence intervals for the median are shown by the vertical gray lines. **C**, Change in platelet count (×10^3^/μL) from day 0 (admission) to day 28 for four patients with a splenial lesion (**left panel**) and six patients without a splenial lesion (**right panel**). Gray circles and lines show individual values; gray squares and lines show medians for each group of patients. Upper and lower 95% confidence intervals for the median are shown by the vertical gray lines. Shaded areas in the figure indicate the laboratory reference range for each measurement.

At admission, the four patients with the splenial lesions compared with the six patients with no apparent abnormality initially had a higher median hematocrit (Figure 3A) (*P* < 0.04), a higher mean serum indirect bilirubin level (Figure 3B) (*P* < 0.03) and a lower mean platelet count (Figure 3A) (*P* < 0.01), as well as a greater decrease in hematocrit over the first three hospital days (Figure 3A) (*P* < 0.003).

No significant differences were found between the four patients with and the six patients without the splenial lesions with respect to mean age, days of fever before admission, parasite count on admission, parasite count after 24 hours of hospitalization (near the time of the initial MRI examination), parasite clearance time or fever clearance time (Table 1). Mean ± SEM serum creatinine did not differ significantly between the groups with and without the splenial lesion either on admission (0.90 ± 0.04 versus 0.78 ± 0.08 U/L; *P* = 0.30) or on day 3 of hospitalization (0.73 ± 0.03 versus 0.68 ± 0.06; *P* = 0.61). Four weeks later, near the time of discharge from the hospital, the groups of patients with and without the splenial lesions did not differ significantly with respect to either median hematocrit (Figure 3A) (*P* = 0.61) or mean platelet count (Figure 3A) (*P* = 0.24).

## Discussion

Each of these 10 patients had acute uncomplicated falciparum malaria, was fully conscious (Glasgow coma score of 14–15) and had no abnormality detected by standard neurologic examination. In 4 (40%) of the 10 patients, a distinctive symmetrical midline lesion in the central splenium of the corpus callosum was found at admission (Figure 1), which is evidence of a combination of tissue increased water content and diffusion restriction. T2FLAIR images showed that the lesion was hyperintense as a result of prolonged tissue T2 relaxation, an indicator of increased tissue water content. In acute phase diffusion-weighted images, the lesion was also hyperintense, both when compared with more lateral portions of the splenium or to the genu of the corpus callosum and when compared with the same area four weeks later (Figure 1), a sign of transient diffusion restriction. Notably, almost half the children in Malawi with cerebral malaria had increased T2 signal intensity changes in the corpus callosum, usually with diffusion-weighted image abnormalities, and predominantly involving the splenium.^15^ Lesions in the splenium of the corpus callosum have been reported in three adults with cerebral malaria,^20^–^22^ and had resolved in the two patients who were re-examined, 36 days^20^ and 4 months later.^21^

Similar changes in the central splenium of the corpus callosum have been described in other, non-malarial disease states,^20^,^23^,^24^ including temporal lobe epilepsy, trauma (shear injury), alcoholism with vitamin deficiency (Marchifava-Bignami), primary demyelination, and encephalitis, but the MRI appearance and time course in our patients most closely resembles high altitude cerebral edema^25^ and may share common pathophysiologic mechanisms. Observational and experimental studies of high-altitude cerebral edema suggest that the primary etiology is hypoxemia, which leads to tissue oxidative stress, endothelial cell injury, and spreading of endothelial cell junctions with blood–brain barrier opening, resulting in a combination of increased tissue water content from vasogenic interstitial edema and of tissue diffusion restriction.^26^,^27^ Hypoxemia in high-altitude cerebral edema may also stimulate a variety of cellular and molecular responses that affect tissue energy metabolism and alter endothelial permeability via vascular endothelial growth factor, oxygen free radicals and upregulation of nitric oxide production.^26^,^27^

In falciparum malaria, sequestration of parasitized erythrocytes in the cerebral microvasculature may produce tissue hypoxia by reducing the oxygen carrying capacity of the parasitized erythrocytes and decreasing oxygen delivery by slowing the passage of erythrocytes. Capillary obstruction or occlusion may cause ischemia and reduced delivery of vascular water, oxygen, and glucose. All of these potential consequences of parasitic sequestration could impair cellular energy metabolism and lead to reduced energy-dependent transmembrane water movement and axonal transport.^28^ The proportional decrease in X-, Y-, and Z-axis ADC measurements in the central splenium of the corpus callosum of our patients suggests a generalized reduction in cellular water motion, including axonal transport. The degree of ADC reduction of approximately 50% seen in our patients is often associated with permanent tissue damage in cerebral ischemia but the observed transient changes seem to have been of insufficient severity to trigger apoptosis and cellular autolysis.

These general considerations do not specifically explain the involvement of the central splenium of the corpus callosum. These focal changes may be related to the vascular anatomy of the splenium where small arterioles penetrate the corpus callosum directly from large arteries, making them more likely to dilate abnormally with increased local blood flow, blood volume, and intra-arterial pressure.^29^ The splenium is also the only region of the corpus callosum supplied by the posterior circulation, which may be more prone to transient opening of the blood–brain barrier.^30^ The microscopic anatomy of the dense myelinated axons in relation to the capillary density in the splenium may also play a role in producing relatively high local tissue hypoxia because of increased oxygen diffusion distances (or relative barriers to diffusion such as the myelin sheath or oxygen steal by oligodendrocytes along the oxygen diffusion pathways).^31^

At admission, the four patients with the splenial lesions had a higher median hematocrit than the six patients without splenial lesions (Figure 3A) (*P* < 0.04), a difference that would increase blood viscosity, reduce flow, and favor sequestration. In addition, the higher mean indirect bilirubin (Figure 3B) (*P* < 0.03) initially in the four patients with splenial lesions relative to the remaining six patients is evidence of increased hemolysis at admission. Subsequently, during the first three days of hospitalization, the median hematocrit decreased more rapidly in the group with the splenial abnormality (Figure 3A) (by 7% versus 1%; *P* < 0.02), despite effective antimalarial treatment. The increased hemolytic activity in the patients with the splenial lesion occurred in conjunction with marked thrombocytopenia: the median platelet count on admission was 48,500 × 10^3^/μL compared with 129,000 × 10^3^/μL in the remaining patients (Figure 3C) (*P* < 0.02).

Earlier reports from Kenya and studies of patients with acute falciparum malaria in northwestern Thailand have identified platelet-mediated clumping or autoagglutination in *P. falciparum* isolates.^32^–^34^ In Thailand, the agglutination phenotype was found in approximately half of the isolates but, notably, was present in 100% of patients with cerebral malaria.^32^ Autoagglutination is believed to involve a platelet-mediated ligand-receptor interaction, possibly mediated by CD36 acting as a receptor for the *P. falciparum* erythrocyte membrane protein 1 on parasitized erythrocytes.^33^,^35^–^37^ In our patients, increased sequestration and destruction of erythrocytes and platelets in platelet-mediated autoagglutinates may have contributed to the severity of the hemolysis and thrombocytopenia, as well as to the microvascular obstruction underlying the lesions in the splenium of the corpus callousum. The power of our study may have been insufficient to detect other differences between those with and without splenial lesions.

The consequences of splenial lesions similar to those shown in Figure 1 would not be evident by standard neurologic examination. The various disconnection syndromes that are produced by damage to the axons passing through the splenium include varieties of alexia, tactile anomia, apraxia, dysgraphia, and other deficits.^38^–^40^ Specialized testing is needed to detect these conditions but detailed assessment of neurocognitive functioning is difficult in acutely ill patients with cerebral malaria and is seldom included in clinical evaluation. In an exceptional study, specialized somatosensory examination of 20 children in Ghana with a recent history of cerebral malaria identified tactile discrimination deficits.^41^ A strong negative correlation (R = −0.72) was found between coma duration and tactile discrimination. The authors concluded on clinical grounds that impaired integrity of axonal tracts in the corpus callosum was likely to be responsible. We plan to include detailed clinical neurocognitive evaluation in future neuroimaging studies of patients with malaria.

The clinical presentation of the neurologic abnormalities associated with falciparum malaria differs between children in Africa and adults in Southeast Asia.^42^ Although cerebral malaria occurs in both populations, children in Africa living in malaria-endemic areas have a much higher incidence of seizures and persistent neurocognitive impairment in survivors is increasingly recognized.^1^,^6^,^42^–^44^ In Southeast Asia, cerebral malaria in adults with no antimalarial immunity often occurs in association with multi-system organ failure but many adult survivors of severe malaria seem to make a full neurologic recovery, at least as judged by standard clinical neurologic evaluation.^10^,^45^ The means whereby *P. falciparum* can produce severe but potentially reversible neurologic complications are still uncertain but accumulating evidence supports axonal injury as at least one pathologic mechanism.^7^,^10^,^28^,^46^

This MRI study of adults with no anti-malarial immunity in Southeast Asia is too small to provide a meaningful estimate of the prevalence of ischemic lesions affecting the brain in patients with acute malaria. In our patients, after effective antimalarial therapy, repeat magnetic resonance studies at the end of the four-week hospitalization found the lesions wholly resolved. We have no grounds for conjecture about the likelihood of resolution of such lesions in the absence of antimalarial therapy or with treatment less effective than prompt administration of the potent antimalarial drugs used in our study. Nonetheless, a large number of persons potentially could be affected. In Southeast Asia, nearly one billion persons are now exposed to malaria and 25% of the world's clinical attacks of malaria occur in this region.^47^–^49^ Episodes of uncomplicated falciparum malaria may be an unrecognized source of neurologic disease and disability in affected populations, both in Southeast Asia and globally.

## Figures and Tables

**Table 1:** Clinical characteristics of patients*

Variable	Age (years)	Days of fever before admission	Parasite count on admission (no./μL)	Parasite count at 24 hours (no./μL)	Parasite clearance time (hours)	Fever clearance time (hours)	Splenial lesion on initial T2FLAIR
	22	3	588,000	36,800	63	94	+
	30	6	9,200†	896	58	48	+
	34	3	50,700	6,720	49	18	+
	23	3	708,000	104,000	94	80	+
							
Mean	27	3‡	118,000§	12,300§	66	64	
SEM	3	1–6	4,300–3,240,000	450–338,000	10	6–114	

	24	30	15,300	48	31	20	−
	45	3	112,000	17,800	79	154	−
	26	5	38,600	95	48	16	−
	18	5	78,800	120	44	16	−
	39	3	3,580	54	81	12	−
	18	3	104,000	9,920	56	42	−

Mean	28	4‡	35,300§	485§	57	18‡	
SEM	5	−3 to 20	8,580–146,000	30–7,840	8	−15–101	
*P*	0.85	0.36	0.29	0.08	0.48	0.26	

* T2FLAIR = T2-weighted fluid-attenuated inversion recovery. Ranges are 95% confidence intervals.

† 128 *Plasmodium vivax*.

‡ Median.

§ Geometric mean.
